# Multisolvent metabolite profiling of coffee waste by UHPLC-HRMS/MS and molecular networking

**DOI:** 10.1016/j.csbj.2025.10.060

**Published:** 2025-10-30

**Authors:** Surachet Soontontaweesub, Rungvigrai Lertsuwan, Thapanee Pruksatrakul, Atchara Paemaee, Verawat Champreda, Navadol Laosiripojana

**Affiliations:** a The Joint Graduate School for Energy and Environment (JGSEE), King Mongkut's University of Technology Thonburi, Prachauthit Road, Bangmod, Bangkok 10140, Thailand; b Biorefinery Technology and Bioproducts Research Group, National Center for Genetic Engineering and Biotechnology (BIOTEC), 113 Thailand Science Park, Phaholyothin Road, Khlong Luang, Pathumthani 12120, Thailand; c Food Biotechnology Research Team, Functional Ingredients and Food Innovation Research Group, National Center for Genetic Engineering and Biotechnology (BIOTEC), 113 Thailand Science Park, Phaholyothin Road, Khlong Luang, Pathumthani 12120, Thailand

**Keywords:** Coffee wastes, Green beans, Spent coffee grounds, Mass spectrometry, Molecular networking

## Abstract

Coffee processing wastes, including defected green beans (GB) and spent coffee grounds (SCG), are underutilized by-products rich in bioactive compounds with promising applications in nutraceuticals and cosmetics. This study profiled metabolites from Arabica and Robusta GB and SCG using five solvents of varying polarity (ethanol, ethyl acetate, toluene, xylene, and hexane). Quantification of chlorogenic acid and caffeine was performed using HPLC-DAD, while comprehensive metabolite profiling was conducted via UHPLC-HRMS/MS and GC-MS. Principal Coordinate Analysis (PCoA) based on Bray-Curtis distance was applied to provide an unsupervised overview of variation according to solvent polarity and raw material type, while a supervised Random Forest (RF) model was used to assess classification performance and group-level consistency. Non-polar solvents tended to recover fatty acids and sterols, especially from SCG, whereas polar solvents such as ethanol extracted higher amounts of hydrophilic antioxidants, including chlorogenic acid from GB. Molecular networking (MN) visualized structurally related metabolite clusters and illustrated solvent- and material-associated distributions. Overall, these findings indicate that extraction method and raw material origin shape metabolite diversity and functional potential in coffee waste materials. The combined use of MN, multivariate statistics, and machine learning offers a complementary strategy for chemical mapping and provides a framework to guide future valorization of coffee by-products, while additional bioactivity validation will be required to establish specific applications.

## Highlights

● First mapping links coffee waste profiles to functional metabolite classes.
● Molecular networking unveiled uncharted metabolite clusters in coffee wastes.
● UHPLC-HRMS/MS exposed a hidden spectrum of bioactives across the polarity range.

## Graphical Abstract

**Figure:**
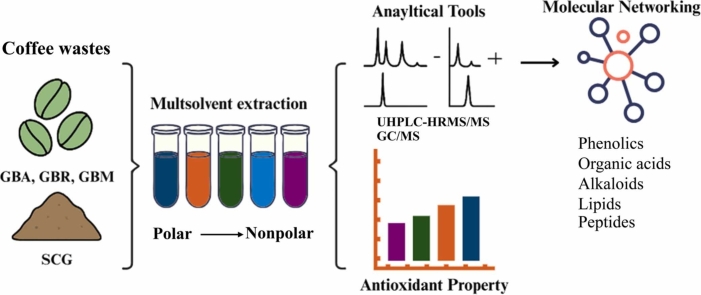

## 1 Introduction

Coffee is the most widely consumed beverage in the world. Processing of coffee generates various by-products and wastes at different stages, including harvesting on farms, roasting in processing facilities, and brewing in coffee shop [1], [2]. Coffee waste produced every year is over 10 million tons, and about half of the wastes is improperly disposed and thus ends up contributing to environmental pollution, with generation of harmful greenhouse gases such as methane and CO_2_ [3]. Notably, approximately 50 kg of defective GB are obtained from one ton of freshly harvested beans, primarily due to imperfections in size, shape, or color during sorting [4]. Additionally, around 45–50 kg of SCG are generated from every 100 kg of roasted coffee processed in cafés and industrial setups [5], [6]. The situation has prompted increasing sustainable alternatives for coffee waste utilization to minimize environmental impacts and create economic value.

Both SCG and GB are rich in bioactive compounds (caffeine, chlorogenic acid, and antioxidants) with the potential to be used in a variety of industries, including food, cosmetics, and pharmaceuticals [5]. Utilization of these by-products as raw materials for functional ingredients is considered a promising way of valorization. Extracts from SCG, with their high levels of phenolic compounds and coffee oil, are used as additives in food, in cosmetics to promote skin health, and in supplements owing to their antioxidant properties [7]. In contrast, GB are richer in chlorogenic acid, which is partially degraded during the roasting step as reflected in its low levels in SCG. Chlorogenic acids provide anti-inflammatory and antioxidant benefits, enhancing the health value of fortified food products [8]. Other bioactive compounds in coffee wastes include a variety of bioflavonoids (e.g., catechins, epicatechins) and diterpenes, which contribute to the complex profiles of coffee metabolites with diverse bioactivities with potential applications across different fields [9], [10]. Various methods have been investigated for extraction and isolation of bioactive compounds from coffee wastes. These include the use of solvent extraction, subcritical water, and supercritical CO_2_ [11], [12], [13]. The abundance of each metabolite varies according to the types of raw materials, crop species, coffee processing, and extraction methods.

Molecular Networking (MN) serves as a data analysis framework that facilitates the organization and interpretation of complex MS/MS datasets. When integrated with mass spectrometry, MN enhances the ability to detect patterns among metabolites and is useful in various fields such as metabolomics, pharmaceutical screening, and food quality evaluation. The technique works by linking spectra with similar fragmentation profiles, which often correspond to structurally related compounds. These relationships are visualized as networks, where each node represents a distinct ion spectrum and edges indicate spectral similarity. This approach facilitates the grouping of structurally related metabolites by spectral similarity, allowing the propagation of annotations from known to unknown features. Such network-based visualization has been widely applied in metabolomics for chemical annotation and prioritization of novel compounds [14], [15].

The application of MN to analyze interactions of complex metabolites in bio-wastes based on structural clustering of the compounds in plant extracts for nutraceutical and cosmetic applications has been demonstrated [16]. This led to the establishment of precise profiles of active metabolites in various raw materials, including fermented teas, medicinal herbs, and coffee-processing by-products [17], [18], [19]. In addition, MN-based approaches have also been used to demonstrate synergistic interactions among bioactive substances with better efficacy in applications like skincare or nutritional supplements [20], [21]. However, MN has not yet been applied to the investigation of complex bioactive metabolites and theirs relationship to bioactivities in coffee wastes. Such an application could help clarify the relationships between extraction methods, metabolite profiles, and biological activities, thereby supporting the further utilization of coffee wastes in nutraceutical, cosmetic, and personal care products.

This study aims to advance understanding on utilizing coffee wastes as valuable resources by analyzing their bioactive compounds and exploring how MN can enhance extraction methods. The complexities of metabolites in Arabica and Robusta GB and SCG were explored using solvent extraction with varying polarities and analyzed by liquid and gas chromatography techniques to cover the full range of hydrophilic to lipophilic compounds. The antioxidant activity of the extracts was also assessed. MN was then applied to understand the relationships between metabolite profiles and activities of coffee extracts obtained from coffee wastes differing in type, crop species, and extraction methods. The information provides a platform for the selection of raw materials and processing methods for transforming coffee wastes into high-value functional products with potential for industrial applications.

## 2 Material and methods

### 2.1 Raw materials and reagents

Four types of coffee waste materials were used in this study: (1) green bean Arabica (GBA), (2) green bean Robusta (GBR), (3) green bean mix (GBM; Arabica: Robusta = 70:30), and (4) spent coffee grounds (SCG) derived from the GBM. All coffee samples were obtained from a local coffee-processing facility (Bangkok, Thailand) and represented typical commercial grades rather than defective beans. SCG originated from espresso preparation using the GBM, which was roasted to a dark roast degree, ground to a fine particle size suitable for espresso brewing and extracted with a commercial espresso machine. The materials were dried at 70 °C for 24 h to reduce moisture content and milled to a particle size of 0.1–1 cm using a mass collider. Ethanol, ethyl acetate, toluene, xylene, and hexane of analytical grade were purchased from Sigma-Aldrich (St. Louis, MO, USA). Reagents for antioxidant assays, 2,2-diphenyl-1-picrylhydrazyl (DPPH) and 2,2′-azino-bis(3-ethylbenzothiazoline-6-sulfonic acid) (ABTS), were obtained from Merck (Darmstadt, Germany). All chemicals used were of analytical grade or higher.

### 2.2 Coffee waste extraction

Dried coffee samples (10 g) were placed in a cellulose thimble inside a Soxhlet extractor. Sequential extraction with each of the five different solvents was performed to obtain different fractions. Each Soxhlet extraction was conducted for 6 h, with the condenser maintained at the boiling point of the respective solvent. Following each 6 h extraction, the solvent was removed by rotary evaporation under reduced pressure (40–50 °C, 150 mbar), and the extract was dried to constant weight. Each extraction was conducted in triplicate, and the results were averaged to ensure data consistency and statistical reliability. The extraction yield (%) was calculated based on the weight of the dried extract relative to the initial mass of coffee material, and the resulting extracts were stored at −20 °C until further use.

Extraction yield (%) = m / M × 100,

*where m* = mass of dried extract (g), *M* = initial mass of coffee material (g)

### 2.3 Quantitation of coffee metabolites

The coffee extracts (10 mg) were dissolved in 1 mL of methanol, vortexed for 1 min, and centrifuged at 10,000 rpm for 10 min. The supernatant was filtered through a 0.22 μm PTFE syringe filter prior to injection. The phenolic compounds in coffee extracts were quantified using HPLC-DAD (Ultimate 3000SL, Dionex, USA) with an Atlantis-C18 column (4.6 × 250 mm, 5 µm) maintained at 40 °C. Elution was carried out at 1 mL/min with a binary mobile phase consisting of 0.4 % formic acid in water (A) and methanol (B), following a 25-minute gradient: 0–20 % B (0–5 min), 40 % B (10 min), 70 % B (17–23 min), and reaching 100 % B at 25 min. Detection was set at 280 nm to monitor caffeine and chlorogenic acid. All analyses were conducted in triplicate, with results expressed as mean ± SD.

### 2.4 Antioxidant activity assays

Coffee extracts were initially dissolved in DMSO and diluted with methanol before being tested with the appropriate DPPH and ABTS reagents at 517 nm and 734 nm, respectively, following established methodologies [20], [22]. Radical scavenging activity was calculated using the equation:

% inhibition = (1 – *A*_sample_ / *A*_control_) × 100,

*where A*_sample_ is the absorbance of the sample-treated solution and *A*_control_ is the absorbance of the blank solution.

The results were expressed as IC50 values, representing the extract concentration required to inhibit 50 % of radicals, with data reported as mean ± standard deviation from triplicate experiments.

### 2.5 Sample preparation for MS analysis

#### 2.5.1 Gas chromatography-mass spectroscopy (GC-MS)

Coffee extracts (15 mg) were dissolved in 1 mL hexane and centrifuged at 10,000 rpm for 20 min. The supernatant was concentrated and freeze-dried at low temperature for 48 h. A portion of the dried sample (10 mg) was derivatized with 20 mg/mL methoxyamination reagent at 37 °C for 2 h, followed by the addition of 70 µL MSTFA and further incubation at 37 °C for 30 min (1100 rpm). After centrifugation at 2000 rpm for 20 min, the clear supernatant was transferred to chromatographic vials.

GC-MS was performed using a QP2020 NX (Shimadzu, Japan) with an SH-Rxi-5Sil MS column (30 m × 0.25 mm × 0.25 µm). The oven was held at 70 °C for 4 min, ramped to 300 °C at 8 °C/min, and held for 10 min. Helium served as the carrier gas at 1 mL/min. The ion source and interface temperatures were 230 °C and 280 °C, respectively. Spectra were recorded in the 35–800 *m/z* range and matched against the NIST17 and in-house libraries. Retention indices were determined using C10–C30 n-alkanes (Restek, Bellefonte,OA). All samples were analyzed in triplicate.

#### 2.5.2 Ultra-high-performance liquid chromatography-high resolution tandem mass spectrometer (UHPLC-HRMS/MS)

Coffee extracts were prepared by dissolving samples in 75 % v/v methanol, centrifuging at 10,000 × g for 15 min at 25 °C, and filtering through a 0.22 µm PTFE membrane prior to analysis. Chromatographic separation was performed using a Vanquish UHPLC system coupled with a Q Exactive™ HF-X Quadrupole-Orbitrap MS (Thermo Scientific, Waltham, USA) and a Hypersil GOLD™ C18 column (100 × 2.1 mm, 1.9 µm, 40 °C). The mobile phase consisted of 0.1 % formic acid in water (A) and 0.1 % formic acid in acetonitrile (B), with gradient elution over 25 min at a flow rate of 400 µL/min. Mass spectrometry was performed in positive and negative ESI modes, with MS1 resolution at 120,000 and data-dependent MS2 (dd-MS2) across a 100–1500 *m/z* range. Each sample was analyzed in triplicate, and the resulting data were used for statistical analysis. To ensure data quality, pooled QC samples were injected at the beginning and throughout the analytical sequence, blanks (75 % methanol) were included to monitor carryover, injection order was randomized, and signal drift was evaluated and corrected during preprocessing.

### 2.6 Compound annotation-based molecular networking

Raw data from UHPLC-HRMS/MS and GC-MS were converted into mzXML format using MSConvert (ProteoWizard, Palo Alto, CA) for compatibility with downstream tools. UHPLC-HRMS/MS data were processed in MZmine (v4.3.0) using feature extraction, deconvolution (ADAP wavelet), isotopic grouping, alignment, and gap filling [23]. The resulting.mgf and quantification tables were submitted to GNPS for Feature-Based Molecular Networking (FBMN) [14], based on cosine similarity (≥0.70), and visualized in Cytoscape (v3.10.2) [24]. For compound annotation, UHPLC-HRMS/MS features were analyzed using SIRIUS (v4.6.1) with CSI:FingerID and ZODIAC, and classified via ClassyFire ontology [25]. Spectra from all raw-material extracts (GBA, GBM, GBR, and SCG) were merged in a single GNPS workspace to visualize solvent-dependent metabolite variation within a unified molecular network and to enable structural comparison among raw materials.

GC-MS data were also converted with MSConvert, then processed using GC-MS EI Data Analysis for spectral deconvolution. The processed spectra were submitted to GNPS for network generation based on spectral similarity, and the final networks were visualized with metadata overlays to reveal compound distribution patterns across extraction solvents and sample types.

### 2.7 Statistical analysis

All experiments were conducted in triplicate unless otherwise specified, and results are presented as mean ± standard deviation (SD). Differences among coffee raw materials and solvents were evaluated using two-way ANOVA followed by Tukey’s HSD post hoc test (p < 0.05), performed in R (v4.4.1). The UHPLC-HRMS/MS and GC–MS data were processed following the FBMN-STATS protocol [2]. Raw mass spectrometry data were pre-processed through blank subtraction, missing value imputation, normalization to total ion current (TIC), and autoscaling prior to statistical evaluation. Multivariate analyses were performed using principal coordinate analysis (PCoA) based on Bray–Curtis distance was used to visualize sample clustering, and statistical significance was tested using PERMANOVA with 999 permutations via the adonis() function (vegan package). Random Forest (RF) classification was conducted using the rfPermute package. Parameters included ntree = 500, mtry = 95, and num.rep = 500. Balanced sampling (balancedSampsize()) was applied to address class imbalance. Model performance was evaluated using the out-of-bag (OOB) error rate, and feature importance was assessed via mean decrease in accuracy (MDA) and Gini index (MDG). All analyses were performed using the FBMN-STATs web app (https://fbmn-statsguide.gnps2.org) and RStudio (v2024.12.0+467), enabling integrative visualization of group separation, metabolite distributions, and model performance.

## 3 Results and discussion

### 3.1 Extraction of metabolites from coffee wastes

**Fig. 1:**
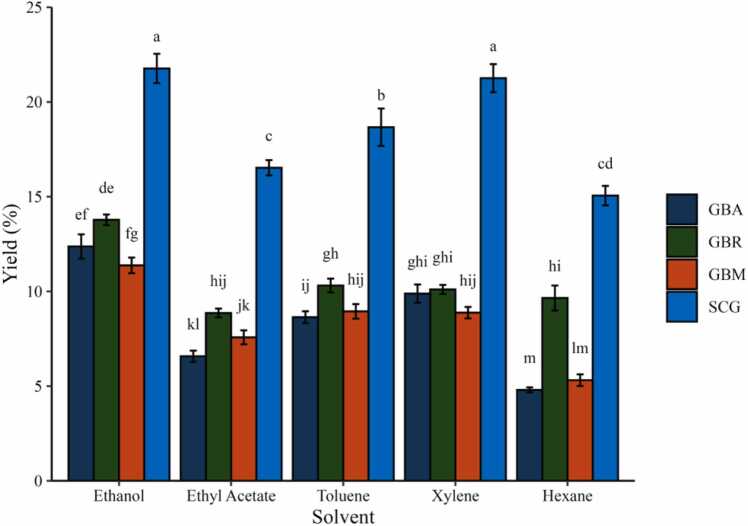
Extraction yields (%) of different coffee materials obtained using various solvents. Values represent mean ± standard deviation (n = 3). Different letters above the bars indicate statistically significant differences (p < 0.05) according to two-way ANOVA followed by Tukey’s HSD test. Abbreviations: GBA = green bean Arabica; GBR = green bean Robusta; GBM = green bean mixed; SCG = spent coffee grounds.

The extraction yields of coffee materials were notably affected by the type of solvent used, as illustrated in Fig. 1. Results are expressed as mean ± SD, and significant differences were determined by two-way ANOVA followed by Tukey’s HSD test (p < 0.05). Among the coffee materials examined, SCG generally exhibited the highest extraction yields across all solvents, with ethanol producing the highest recovery at 21.77 %. This was followed by xylene (21.26 %) and toluene (18.67 %). In comparison, GBA and GBM produced lower yields, particularly in the non-polar solvents such as hexane, which resulted in only 4.8 % for GBA and 5.31 % for GBM. These differences suggest that both solvent polarity and the physicochemical characteristics of the raw materials contribute to extraction efficiency. It should also be noted that the higher yields observed in SCG may partly reflect structural modifications introduced by roasting and brewing, which increase porosity and solvent accessibility in addition to altering intrinsic chemical composition [26], [27]. During roasting, extensive Maillard reactions, caramelization, and pyrolysis occur, leading to profound shifts in the coffee matrix. These thermal reactions transform carbohydrates, proteins, and chlorogenic acids into a variety of newly formed compounds such as melanoidins, furans, pyrroles, and pyrazines, which are absent in green coffee. Such reactions not only reduce native hydrophilic antioxidants but also generate novel lipid-soluble and nitrogen-containing species that contribute to the distinct physicochemical and extraction characteristics of roasted coffee [28], [29].

Building on this general trend, differences among solvents of varying polarity provide further insight into the recovery of specific metabolite classes. Ethanol, a polar solvent, generally yielded higher recoveries across all coffee materials due to its ability to dissolve a broad range of hydrophilic and semi-polar bioactive compounds, such as chlorogenic acids, flavonoids, and phenolic compounds. In this study, ethanol extraction of SCG produced the highest yield (21.8 %), exceeding the 13.1 % reported by Somnuk et al. (2017) under comparable conditions [30]. This discrepancy may reflect both differences in extraction parameters and roasting-induced matrix changes in SCG that facilitate solvent penetration. Ethanol also consistently extracted more material from GB compared to other solvents, highlighting their richness in hydrophilic compounds. This observation is consistent with previous reports indicating that green coffee beans are abundant in chlorogenic acid derivatives, flavonoids, and nitrogen-containing compounds, which exhibit a strong affinity for polar protic solvents such as ethanol due to hydrogen-bonding interactions and partial miscibility with water.

In contrast, non-polar solvents preferentially recover fatty acids, sterols, and diterpenes, reflecting their higher solubility for lipophilic compounds derived from coffee lipids and waxes. Similar solvent-dependent extraction behavior has been reported for green coffee matrices, where ethanol and aqueous-ethanol systems yielded higher levels of phenolic and nitrogenous compounds than less-polar organic solvents [7], [9]. Among the GB samples, GBR yielded slightly higher values than GBA, particularly in ethanol and non-polar solvents, which is consistent with prior reports of higher lipid and phenolic contents in Robusta compared with Arabica [7]. GBM produced intermediate yields, reflecting its blended composition.

Moderate yields were observed for solvents with intermediate polarity, such as ethyl acetate and toluene. For instance, toluene extraction of SCG resulted in an 18.67 % yield, compared with 8.6 % for GBA and 8.9 % for GBM. This pattern suggests that semi-polar compounds, such as lipophilic phenolics or caffeine derivatives, are more accessible in SCG owing to matrix changes introduced by roasting and brewing. In contrast, non-polar solvents, such as hexane, exhibited low extraction efficiencies when applied to green coffee materials due to their limited ability to solubilize polar compounds. However, SCG yielded considerably higher values with hexane (12–15 %), consistent with the retention of lipophilic compounds after brewing. For example, Efthymiopoulos et al. (2018) reported hexane extractions from SCG achieving lipid yields up to 14 %, highlighting the presence of triglycerides and neutral lipids [32]. Similarly, Somnuk et al. (2017) optimized hexane-based extraction and reported yields up to 14.7 %, confirming the richness of SCG in oils and fatty acids [30]. The present observation of a 15.06 % yield using hexane further underscores its lipid-rich nature and chemical complexity.

### 3.2 Quantitation of active compounds

**Fig. 2:**
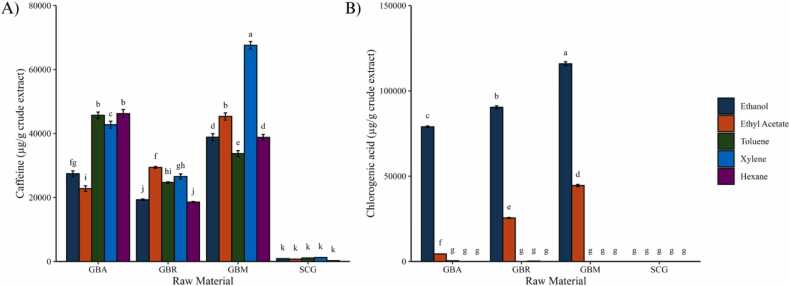
Concentrations of bioactive compounds in coffee extracts: (A) caffeine and (B) chlorogenic acid obtained from different coffee raw materials using different solvents. Data are expressed as mean ± SD (n = 3). Different letters above the bars indicate statistically significant differences according to two-way ANOVA followed by Tukey’s HSD test (p < 0.05). GBA = green bean Arabica; GBR = green bean Robusta; GBM = green bean mixed; SCG = spent coffee grounds.

The quantification of caffeine and chlorogenic acid in coffee extracts revealed clear variation across both coffee materials and extraction solvents, as shown in Fig. 2. Results are expressed as mean ± SD, and statistical significance was evaluated by two-way ANOVA followed by Tukey’s HSD test (p < 0.05). GBM exhibited the highest caffeine content, with statistically significant differences, particularly in xylene extracts, followed by GBA. Interestingly, GBR showed comparatively lower caffeine levels, although Robusta is generally reported to contain higher caffeine concentrations than Arabica [7], [9]. Such differences may arise from variability in cultivation origin and post-harvest processing among commercial Robusta batches, which affect alkaloid biosynthesis and retention. SCG contained substantially lower caffeine content across all solvents, which was statistically significant compared with GB, likely reflecting both depletion of soluble caffeine during brewing and structural modifications from roasting that reduce matrix accessibility [33].

In contrast, chlorogenic acid was most efficiently extracted using ethanol across GBM and GBR extracts, showing significantly higher concentrations than GBA. Ethyl acetate provided moderate recoveries, while non-polar solvents and SCG contained only minor amounts, consistent with the polar nature of chlorogenic acids and their depletion during brewing. The statistical grouping indicated by different letters in Fig. 2B confirms that ethanol extracts of GBR and GBM were significantly higher than GBA (p < 0.05). These results agree with prior studies reporting that Robusta generally contains higher levels of chlorogenic acids than Arabica, contributing to its stronger antioxidant potential [7]. Taken together, these findings highlight that ethanol is particularly effective for recovering hydrophilic compounds such as chlorogenic acids, whereas non-polar solvents have limited utility. Importantly, extraction outcomes are not solely determined by solvent polarity but are also influenced by raw material properties. The observed differences between Arabica and Robusta, as well as the mixture, may partly reflect intrinsic compositional variation but also structural and physicochemical factors such as cell integrity, roasting history, and matrix porosity, which affect solvent penetration and metabolite accessibility [33]. Thus, optimization of bioactive recovery requires consideration of both solvent polarity and the physicochemical attributes of the coffee material. Previous studies have also reported varying phenolic recovery yields depending on solvent systems, ranging from 1.2 to 21.8 % with conventional solvents [29], [30] and 2.5–15 % using supercritical CO_2_ extraction methods [34], further supporting the role of extraction conditions in bioactive compound recovery.

### 3.3 Antioxidant properties of coffee crude extracts

**Fig. 3:**
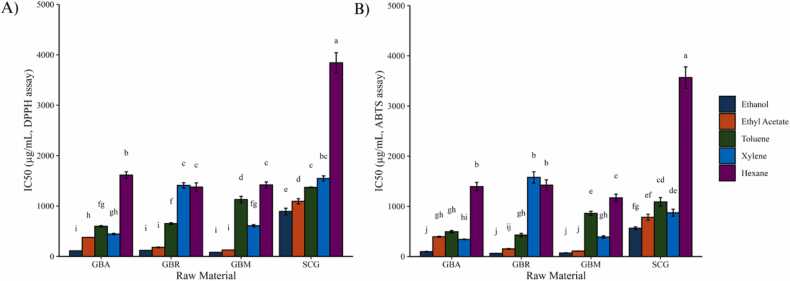
Antioxidant activity of coffee crude extracts expressed as IC₅₀ values (µg/mL) determined by (A) DPPH assay and (B) ABTS assay for different coffee raw materials. Data are presented as mean ± SD (n = 3). Different letters above the bars indicate statistically significant differences according to two-way ANOVA followed by Tukey’s HSD test (p < 0.05). GBA = green bean Arabica; GBR = green bean Robusta; GBM = green bean mixed; SCG = spent coffee grounds.

The antioxidant activity of coffee extracts was assessed using DPPH and ABTS radical scavenging assays, with results expressed as IC50 values in (Fig. 3), where lower values indicate higher antioxidant efficiency. Results are expressed as mean ± SD, and statistical significance was determined using two-way ANOVA followed by Tukey’s HSD test (p < 0.05). Ethanol extracts consistently exhibited the lowest IC50 values across all coffee materials relative to other solvents, with significant differences among solvent groups within each raw material. Among the coffee materials, GBM exhibited the strongest antioxidant activity. For the DPPH assay, GBM ethanol extracts achieved an IC50 value of 81.7 ± 1.0 µg/mL, while for the ABTS assay, the IC50 value was 71.0 ± 4.5 µg/mL, reflecting its richness in extractable antioxidant compounds. Its superior antioxidant performance compared to the individual GBA and GBR is likely associated with differences in bean origin, post-harvest handling, and blending uniformity inherent to commercial coffee processing. Variations in moisture content, drying conditions, or particle size among batches could have enhanced solvent accessibility and facilitated the release of bioactive compounds [9], [35]. GBR also showed high antioxidant activity, with ethanol extracts achieving IC50 values of 120.0 ± 0.6 µg/mL and 64.4 ± 1.5 µg/mL for DPPH and ABTS assays, respectively, which were significantly lower than those of GBA ethanol extracts (p < 0.05). This is in line with previous reports showing that Robusta tends to exhibit higher chlorogenic acid content and antioxidant potential compared with Arabica, consistent with previous reports [7], [36].

In contrast, SCG exhibited the weakest antioxidant activity, with higher IC50 values across both assays. For example, the IC50 values for SCG ethanol extracts were 892.1 ± 65.4 µg/mL in the DPPH assay and 565.0 ± 25.9 µg/mL in the ABTS assay. These values were significantly higher (p < 0.05) than those of GB, confirming its reduced antioxidant potential. This lower activity reflects both the extraction of hydrophilic antioxidants such as chlorogenic acids during brewing and roasting-induced structural changes that increase porosity while reducing residual activity [37], [38], [39]. Overall, solvent polarity significantly influenced antioxidant activity, with ethanol outperforming ethyl acetate and non-polar solvents such as hexane, which exhibited significantly weaker antioxidant effects due to limited extraction of polar compounds (p < 0.05). These findings highlight the importance of both solvent properties and raw material characteristics—including processing-induced structural changes—in shaping antioxidant potential.

### 3.4 Metabolite profiling of coffee crude extracts via mass spectrometry

#### 3.4.1 UHPLC-HRMS/MS

Metabolite profiling of coffee extracts was conducted using UHPLC-HRMS/MS in both negative and positive modes. The data were processed and visualized using GNPS platform tools to explore variations in metabolite profiles. To evaluate the effects of solvent polarity and raw material type, PCoA was applied using Bray–Curtis distance, which is well suited for complex, high-dimensional data. Both ionization modes were employed to ensure broad metabolite coverage. The results showed that the negative mode provided clearer separation among groups compared with the positive mode. However, some technical replicates, particularly GBR samples in the negative mode, exhibited greater within-group variation than between-group differences. This variation may be attributed to minor inconsistencies in extraction or instrumental response [40], [41], possibly combined with subtle differences in particle size or moisture content within the same batch [42], indicating that part of the observed clustering may arise from technical variation as well as solvent-driven effects.

**Fig. 4:**
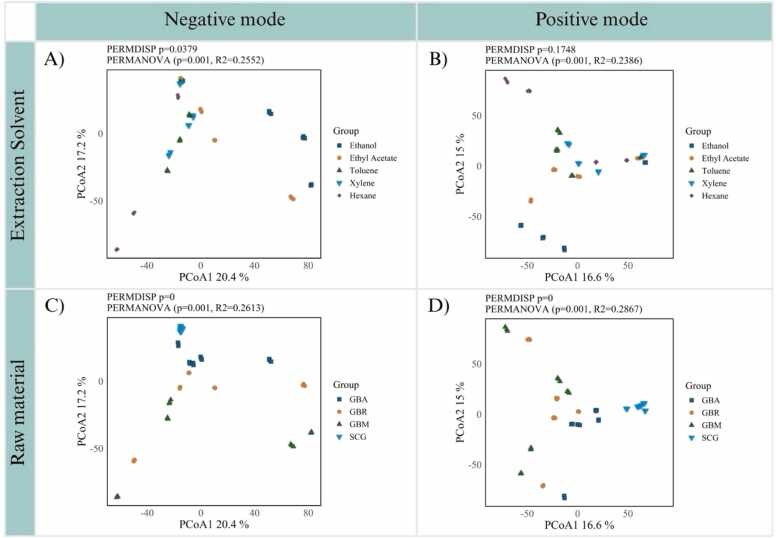
Principal Coordinate Analysis (PCoA) of coffee-extract metabolite profiles. Separation is shown by extraction solvent in A) negative and B) positive modes, and by raw material in C) negative and D) positive modes. PCoA axes indicate the percentage of variance explained. GBA = green bean Arabica; GBR = green bean Robusta; GBM = green bean mixed; SCG = spent coffee grounds.

PCoA based on Bray–Curtis distance provided an unsupervised overview of variation in metabolite profiles across solvents and raw materials (Fig. 4). When considering solvent effects, the negative mode (Fig. 4A) showed clearer separation among metabolite profiles derived from different solvents than the positive mode (Fig. 4B). The PERMANOVA result in the negative mode (R^2^ = 0.2552, p = 0.001) indicates that solvent-dependent extraction profiles accounts for approximately 26 % of the total variance, suggesting a substantial influence of solvent polarity on metabolite composition. Metabolite profiles of samples extracted with toluene and xylene clustered closely together, whereas those extracted with hexane were more dispersed along the second principal component, indicating that metabolite profiles derived from non-polar extractions did not form a cohesive group. This dispersion may reflect heterogeneous recovery of lipophilic compounds by hexane. In contrast, the metabolite profile from ethanol extraction was clearly separated along the first principal component, while the profile from ethyl acetate extraction grouped closer to those derived from non-polar extractions, consistent with its intermediate polarity and partial overlap in extraction patterns. PERMDISP (p = 0.0379) suggests unequal within-group dispersion, explaining partial overlap between profiles derived from toluene and hexane extractions. In the positive mode, PERMANOVA (R^²^ = 0.2386, p = 0.001) indicates that variation among solvent-derived metabolite profiles explains about 24 % of the variance. The weaker group separation, together with the non-significant PERMDISP (p = 0.1748), likely reflects the ionization characteristics of positive mode, which preferentially detects alkaloids, lipids, and other cation-forming compounds that are more broadly distributed across solvent extracts. In contrast, the negative mode provides stronger signals for phenolic acids and chlorogenic acids, thereby enhancing discrimination among solvent-derived metabolite profiles.

In addition to solvent effects, PCoA was used to examine the influence of raw material type to metabolite composition (Figs. 4C and 4D). For raw material effects in the negative mode (Fig. 4C), PERMANOVA (R^2^ = 0.2613, p = 0.001) indicates that variation among metabolite profiles derived from different raw materials explains roughly 26 % of the variance, with SCG forming a distinct cluster apart from GB. This divergence likely reflects compositional shifts in metabolite profiles caused by roasting and brewing, leading to increased lipid residues and thermally derived compounds in SCG [38], [39]. In the positive mode (Fig. 4D), PERMANOVA (R^2^ = 0.2867, p = 0.001) indicates that raw material type accounts for variation in metabolite profiles, highlighting the influence of roasting and brewing in SCG compared with unprocessed GB. SCG profile consistently clustered apart from all GB types, reflecting compositional changes associated with thermal treatment and extraction during brewing [33]. PERMDISP supported significant dispersion differences in both negative and positive modes, suggesting that the observed separation was driven by variation in metabolite composition both within and between raw material groups.

**Fig. 5:**
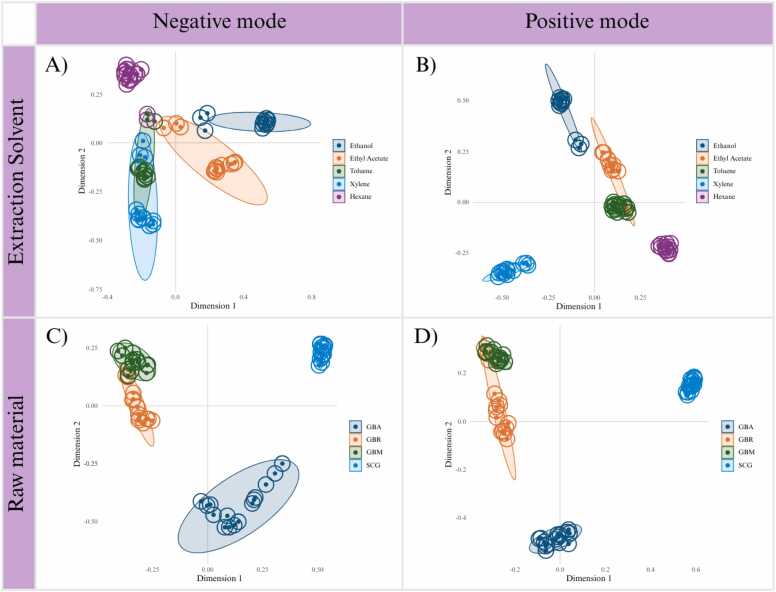
Random Forest (RF) analysis of coffee-extract metabolite profiles. Separation is shown by extraction solvent in A) negative and B) positive modes, and by raw material in C) negative and D) positive modes. Axes represent RF dimensions. GBA = green bean Arabica; GBR = green bean Robusta; GBM = green bean mixed; SCG = spent coffee grounds.

While PCoA provided an unsupervised overview of sample clustering, RF classification was employed to quantitatively assess classification accuracy and identify potential misclassifications (Fig. 5). The RF models were constructed with 500 trees, and performance was evaluated through out-of-bag (OOB) error rates, confusion matrices, and permutation-based significance testing of variable importance. For solvents, the RF plots showed distinct groupings in both ionization modes. In the negative mode (Fig. 5A), non-polar solvents (hexane, toluene, xylene) were clearly separated from polar solvents (ethanol, ethyl acetate). Toluene displayed partial overlap with other non-polar solvents, which corresponded to its lower classification accuracy (83.3 %; Supplementary Table S2). In contrast, ethanol, ethyl acetate, hexane, and xylene were classified with 100.0 % accuracy. In the positive mode (Fig. 5B), separation among solvent groups was still evident, but ethanol and ethyl acetate overlapped more strongly, resulting in a reduced class accuracy of 75.0 % for ethyl acetate (Supplementary Table S4). Overall, solvent classification achieved 96.7 % accuracy in the negative mode and 95.0 % in the positive mode. For raw materials, RF classification showed excellent performance in both ionization modes (Figs. 5C and 5D). SCG consistently formed a tight, well-separated cluster distinct from GB, reflecting compositional divergence due to roasting and brewing [33]. GBA, GBR, and GBM also formed separate classes, with subtle intra-group structure particularly visible in the positive mode (Fig. 5D). Confusion matrices (Supplementary Tables S1 and S3) confirmed perfect classification: all raw material types achieved 100.0 % accuracy with 95 % confidence intervals ranging from 78.2 % to 100.0 %, and the overall accuracy was 100.0 % in both ionization modes.

The robustness of the RF models was further supported by OOB error curves (Supplementary Figures S1–S4). For raw material classification, the OOB error dropped to zero after approximately 50 trees and remained stable across 500 trees, consistent with perfect classification observed in the confusion matrices. For solvent classification, OOB error stabilized below 5 %, mirroring the few misclassifications detected between toluene and ethyl acetate. These results demonstrate that the models converged rapidly and maintained high predictive accuracy, reinforcing the reliability of the classification outcomes.

Collectively, these results demonstrate that the RF models provide robust supervised confirmation of the clustering trends observed in the unsupervised PCoA. Feature importance analysis (Supplementary Figures S5–S8) further enhanced the interpretability of the RF classification by highlighting the variables that most strongly contributed to class separation. The combined evidence from confusion matrices, OOB error rates, and statistical validation indicates that the variation in metabolite composition is partially explained by solvent extraction and raw material type, rather than resulting from random variation. Notably, the consistent and clear separation of SCG from GB underscores the pronounced compositional changes induced by roasting and brewing. Likewise, the solvent-dependent groupings emphasize the central role of solvent polarity in shaping the extraction-derived metabolite profiles of coffee waste.

**Fig. 6:**
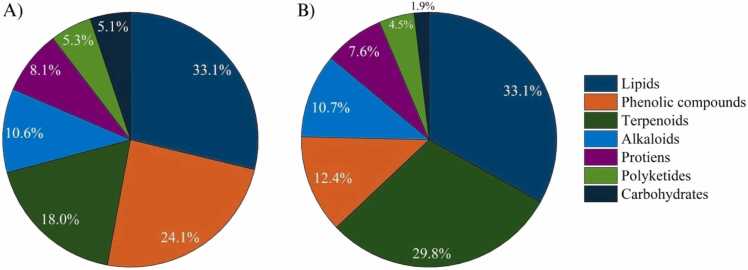
Chemical composition of coffee extracts identified using SIRIUS from UHPLC-HRMS/MS data in A) negative mode and B) positive mode. Compound classes were predicted using the CANOPUS algorithm based on MS/MS fragmentation patterns.

To better understand the chemical nature underlying these separations, metabolite annotation and classification were carried out in SIRIUS based on UHPLC-HRMS/MS data (Fig. 6). Lipids represented the largest annotated class in both ionization modes, reflecting their prevalence in coffee matrices [31,44]. This dominance of lipid-related compounds is consistent with previous reports on roasted coffee, where roasting promotes the accumulation of triglycerides and diterpenes while reducing phenolic acids [44]. In the negative ionization mode, phenolic compounds were more prominently detected. This observation is consistent with established mass spectrometry studies showing that phenolic acids, particularly chlorogenic acids, exhibit stronger ionization efficiency in negative electrospray mode. This arises from the ease of deprotonation of their hydroxyl and carboxyl groups [35]. The enrichment of chlorogenic acids in the negative mode also agrees with earlier metabolomics studies reporting higher phenolic content in green Arabica compared with roasted or Robusta beans [7].

By contrast, the positive mode highlighted polyketides and nitrogenous metabolites, consistent with previous reports showing that positive ESI provides greater sensitivity for alkaloids, diterpenoids, and other lipophilic compounds [43]. These observations reflect differences in ionization behavior rather than extraction efficiency, as recovery rates were not directly quantified. Importantly, the mode-dependent patterns emphasize the complementary roles of negative and positive ionization in expanding chemical coverage of coffee extracts. The negative mode facilitated broader chemical diversity, while the positive mode produced clearer signals for certain dominant metabolite classes. Together, these results demonstrate that integrating both ionization modes enhances metabolome coverage, in agreement with prior studies that recommended dual-mode profiling to capture both phenolic-rich fractions typical of Arabica and lipophilic diterpenes enriched in roasted and Robusta coffees [7,44]. These observed patterns may also be shaped by sample processing factors such as roasting and grinding, which alter porosity and matrix accessibility [45]. While Fig. 6 provides a class-level overview of metabolite distributions, additional resolution is required to identify which individual metabolites drive these differences. To address this, compound-level abundance patterns were examined using heatmaps.

**Fig. 7:**
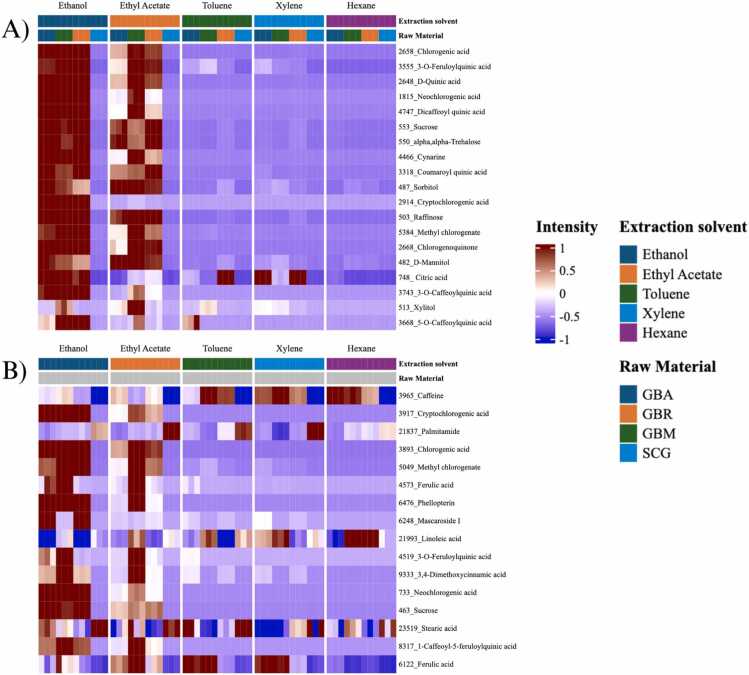
Heatmap of metabolite intensities in coffee extracts analyzed by UHPLC-HRMS/MS in A) negative mode and B) positive mode. GBA = green bean Arabica; GBR = green bean Robusta; GBM = green bean mixed; SCG = spent coffee grounds.

The heatmap in Fig. 7 displays the scaled and centered intensities of metabolites identified from coffee extracts, analyzed by UHPLC-HRMS/MS under negative (Fig. 7A) and positive (Fig. 7B) modes. The hierarchical clustering revealed distinct metabolite patterns influenced by the extraction solvents and raw material types, providing insights into the abundance and variability of both polar and non-polar metabolites. In the negative mode, polar metabolites such as chlorogenic acids, neochlorogenic acid, caffeoylquinic acid, and cryptochlorogenic acid showed relatively high intensities, particularly in extracts obtained using polar solvents such as ethanol and ethyl acetate, especially from GB samples. This apparent enrichment in SCG likely reflects the relative concentration of less-extracted, lipophilic compounds that remain after brewing, such as sterols and diterpenes, rather than an absolute increase. Previous studies have shown that although brewing depletes hydrophilic antioxidants, lipophilic constituents can persist in higher relative amounts due to their low solubility in water and stability during roasting [44].

These results are consistent with established ionization trends, where phenolic acids preferentially respond in negative mode due to their ease of deprotonation and multiple hydroxyl and carboxyl groups [46,47]. In contrast, the positive mode heatmap highlighted nitrogenous and lipophilic metabolites such as caffeine, trigonelline, diterpenes (e.g., cafestol), and lipid–amine conjugates (e.g., N-behenoyl-5-hydroxytryptamide), which appeared more abundant in SCG extracts obtained with non-polar solvents (hexane, toluene, xylene). These findings align with prior metabolomics reports showing that positive ionization enhances detection of alkaloids and diterpenoids in coffee matrices [44]. Moderate signals of these lipophilic compounds were also observed in ethanol and ethyl acetate extracts, suggesting that these solvents can capture a broader range of metabolite classes under the tested conditions. Taken together, these results are consistent with previous research reporting that solvent polarity and matrix structure jointly shape metabolite recoveries. Roasting-induced porosity and lipid retention in SCG alter accessibility and distribution of detected compounds [7], [30]. Overall, the heatmap analysis confirms that solvent polarity and raw material type strongly influence metabolite distributions, while also underscoring that structural changes introduced during roasting and brewing—such as increased porosity and altered matrix accessibility—likely further contribute to the observed separation patterns.

**Fig. 8:**
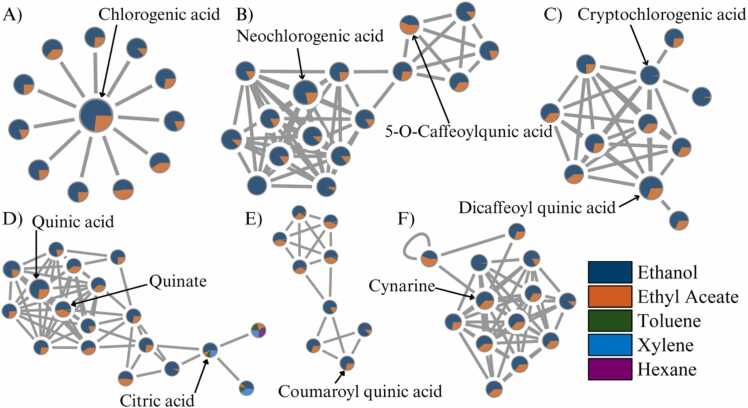
Molecular networking clusters of phenolic and organic acids in coffee extracts analyzed in negative mode.

**Fig. 9:**
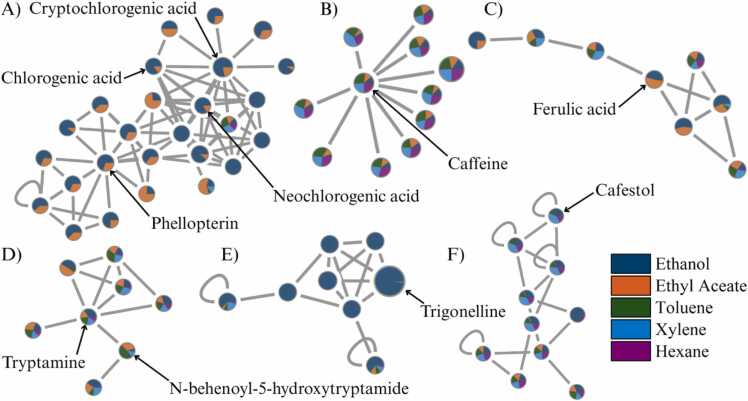
Molecular networking clusters of alkaloids, lipids, and peptides in coffee extracts analyzed in positive mode.

Fig. 8 and Fig. 9 present selected MN clusters from UHPLC-HRMS/MS analysis of coffee extracts, highlighting key metabolite groups detected in negative and positive modes, respectively. These figures demonstrate how ionization mode and solvent polarity influence the annotation and distribution of structurally diverse bioactive compounds in GB and SCG. In negative mode (Fig. 8), the MN reveals a range of structurally related compounds dominated by phenolic acids and small organic acids. The network includes chlorogenic acid and its isomers (cluster A), neochlorogenic acid and 5-O-caffeoylquinic acid (cluster B), and cryptochlorogenic acid together with dicaffeoylquinic acid (cluster C). All of these metabolites share hydroxycinnamate backbones and form dense spectral clusters. Additional subnetworks include quinic acid, quinate, and citric acid (cluster D), coumaroylquinic acid derivatives (cluster E), and cynarine and its related dicaffeoyl esters (cluster F). These compounds were more prominent in ethanol and ethyl acetate extracts, consistent with their polarity. Negative mode thus enhanced detection of metabolites with multiple hydroxyl and carboxyl groups, improving coverage of polar compounds in both GB and SCG.

In positive mode (Fig. 9), the molecular network (MN) highlights metabolite classes less prominent in the negative mode, such as alkaloids, lipophilic compounds, and other nitrogen-containing metabolites. Key annotations include caffeine (cluster B), ferulic acid and its derivatives (cluster C), a group of nitrogen-containing compounds including tryptamine and N-behenoyl-5-hydroxytryptamide (cluster D), trigonelline (cluster E), and cafestol (cluster F). These compounds showed higher relative intensities in extracts obtained with non-polar solvents, reflecting the persistence of lipophilic and nitrogenous metabolites after roasting and brewing [33]. To visualize solvent-dependent variation, MNs were constructed by combining all raw-material spectra within the same framework, and the distribution of metabolites was interpreted primarily in relation to extraction solvent. This approach enabled structural comparison across solvents while recognizing that unroasted beans and roasted/brewed residues differ substantially in chemical composition. The visualization allowed identification of clusters shared across all sample types (e.g., chlorogenic acids) and highlighted solvent-specific enrichment patterns. Importantly, MN provides putative structural groupings rather than definitive compound characterization. Further targeted analyses (e.g., MS/MS validation or quantitative assays) are required to confirm compound identities and assess valorization potential.

Together, the MN analyses highlight the complementary role of dual ionization modes in providing a broader descriptive overview of coffee metabolites. The negative mode facilitated the detection of polar metabolites such as phenolic and organic acids, whereas the positive mode provided additional coverage of nitrogenous, lipidic, and diterpenoid compounds less prominent in the negative mode. These patterns indicate that using both ionization strategies provides a more complete representation of the chemical diversity present in coffee extracts. It should be noted, however, that observed differences between GB and SCG may partly reflect structural and physicochemical changes introduced during roasting and brewing, including altered porosity and solvent accessibility, in addition to intrinsic compositional differences. Rather than implying extraction efficiency or direct valorization potential, the present findings provide a descriptive overview of solvent- and mode-dependent metabolite profiles that can inform future, more targeted studies on the utilization of coffee by-products.

#### 3.4.2 GC-MS

**Fig. 10:**
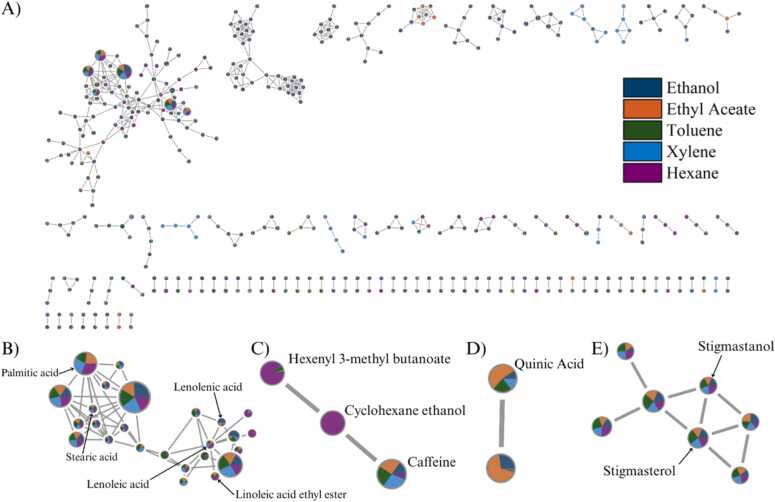
Molecular networking of coffee extracts from GC-MS analysis. (A) Overview of the molecular network colored by extraction solvents. (B–E) Highlighted clusters of key metabolites: (B) fatty acids, (C) aroma compounds, (D) quinic acid, and (E) sterols. Node pie charts represent compound distribution across solvents.

GC-MS-based MN revealed a diverse array of lipophilic metabolites in the coffee extracts (Fig. 10A). The global network contained 419 nodes, with 304 metabolites successfully annotated. Clustering patterns clearly corresponded with chemical classes and extraction solvent polarity.

Non-polar solvents such as hexane and toluene predominantly highlighted clusters enriched in fatty acids and sterols, including palmitic acid, stearic acid, and linoleic acid (Fig. 10B). These lipophilic features were especially prominent in SCG extracts, confirming their richness in coffee oil residues after brewing. In contrast, semi-polar solvents like ethyl acetate and ethanol enabled the extraction of a broader range of compounds. These included volatile esters (e.g., hexenyl 3-methyl butanoate) and polar organic acids such as quinic acid (Fig. 10C and Fig. 10D). Additional sterol-related features, including stigmastanol, were particularly abundant in SCG extracts obtained with hexane and xylene (Fig. 10E). The comparatively weaker recovery of these compounds in GB may reflect the intact cell structures of unroasted beans, which can limit the efficiency of non-polar solvent extraction despite the presence of fatty acids in their composition [48].

The MN approach in this study facilitated the visualization of relationships among compounds that would be difficult to resolve using conventional chromatographic or MS1-based analyzes. Specifically, it enabled the clustering of isomeric compounds such as chlorogenic acid derivatives, dicaffeoylquinic acid, trigonelline, and diterpenes. highlighted solvent-specific extraction patterns and co-occurrence trends unique to GB and SCG. These network-based insights provide a broader structural context and can guide tentative annotation by highlighting spectral similarities, although definitive confirmation requires targeted validation [14], [15].

Compared to previous coffee metabolomics studies, which often used single solvents such as ethanol or methanol and focused mainly on well-known hydrophilic phenolics [7], [33], the present multi-solvent strategy revealed broader coverage, particularly for lipophilic compounds. For instance, while ethanol was effective in detecting chlorogenic acids in GB, non-polar solvents such as hexane and toluene showed stronger associations with sterols, fatty acids, and diterpenes, which have also been reported as prominent constituents of SCG-derived oils in previous studies [30], [31]. Importantly, the MN analysis uncovered several unannotated nodes with high cosine similarity (≥0.7) to known lipid conjugates and diterpenoids, suggesting the presence of potentially novel or poorly characterized compounds. Rather than implying extraction efficiency or depletion directly, these results highlight the value of combining orthogonal extraction and analysis approaches to capture the chemical diversity of coffee by-products.

Collectively, this comprehensive mapping provides a robust platform for prioritizing metabolite classes based on their chemical diversity and bioactivity potential. Ethanol extracts from GB, which contain relatively higher levels of phenolic compounds such as chlorogenic and dicaffeoylquinic acids, suggest possible value for antioxidant-oriented nutraceutical applications. In contrast, lipophilic fractions from SCG, particularly those associated with hexane and toluene extracts, contained dense clusters of sterols, fatty acids, and diterpenoids that are often linked with cosmetic or dermatological relevance in previous studies [10], [19]. This enrichment in SCG is likely influenced not only by its intrinsic chemical composition but also by sample preparation effects. Roasting and brewing disrupt cellular integrity and increase oil accessibility, while simultaneously inducing Maillard and pyrolytic reactions that generate new lipophilic compounds such as diterpenes, fatty acid amides, and melanoidin-associated structures [48,49]. These thermally derived species may further contribute to the complex chemical profile observed in SCG lipid fractions. This lipophilic enrichment aligns with the observed potential of SCG oils for cosmetic and dermatological formulations reported in previous studies [5], [10]. Moreover, the presence of uncharacterized compounds within SCG lipid clusters presents opportunities for novel bioactive discovery, warranting future isolation and functional evaluation.

Overall, the integration of multi-solvent extraction, UHPLC-HRMS/MS, GC-MS, and MN provides a comprehensive overview of the metabolite landscape in coffee by-products and illustrates how solvent polarity and raw material origin shape chemical profiles. By comparing MN insights with antioxidant activity profiles, this study shows that GB extracts obtained with polar solvents–particularly ethanol and ethyl acetate–contained higher levels of phenolic compounds such as chlorogenic and dicaffeoylquinic acids. In contrast, SCG extracts obtained with non-polar solvents such as hexane and toluene showed dense clusters of sterols, fatty acids, and diterpenoids. Importantly, observed differences between GB and SCG should be interpreted cautiously. Roasting, brewing, and grinding alter porosity and solvent accessibility as well as chemical composition, and may therefore contribute to observed separations. Likewise, potential differences among GBA, GBR, and GBM were not statistically confirmed and remain preliminary observations [5], [19], [29].

Finally, while MN allowed structural grouping of metabolites, its limitations include reliance on spectral libraries, lack of quantitative scaling, and underrepresentation of poorly ionizing compounds. The use of PCoA and Random Forest analyses confirmed clustering by solvent and raw material, but rather than identifying definitive markers, they highlighted discriminative features that warrant further validation. Future studies should emphasize targeted bioassays, toxicological profiling, and integrative omics (e.g., metabolotranscriptomics or lipidomics) to validate functionality and support the development of coffee residues as potential sources of bioactive compounds [14], [23].

## 4 Conclusion

This study highlights the potential of GB and SCG as valuable resources for bioactive compounds, including caffeine and chlorogenic acids. Among the tested solvents, ethanol generally yielded higher recovery of hydrophilic antioxidants, particularly chlorogenic acids. By contrast, non-polar solvents such as hexane extracted lipophilic constituents, including fatty acids and sterols, especially from SCG. MN enabled visualization of metabolite clusters across solvent polarities and material types; however, its reliance on spectral similarity and limited annotation coverage remain constraints. The integration of PCoA and Random Forest analyses offered complementary perspectives by highlighting separation patterns associated with solvent and raw material type, and by pointing to discriminant features primarily related to phenolic acids, fatty acids, and nitrogen-containing metabolites. These findings underscore the benefit of combining untargeted metabolomics with machine learning for robust chemical profiling and demonstrate the distinct metabolite enrichment patterns of GB and SCG extracts across solvent systems.

## Author statement

We the undersigned declare that this manuscript is original, has not been published before and is not currently being considered for publication elsewhere.

We confirm that the manuscript has been read and approved by all named authors and that there are no other persons who satisfied the criteria for authorship but are not listed. We further confirm that the order of authors listed in the manuscript has been approved by all of us.

We understand that the Corresponding Author is the sole contact for the Editorial process.

## CRediT authorship contribution statement

**Rungvigrai Lertsuwan:** Formal analysis, Data curation. **Thapanee Pruksatrakul:** Visualization, Methodology, Formal analysis. **Surachet Soontontaweesub:** Writing – original draft, Validation, Methodology, Investigation, Formal analysis, Data curation. **Verawat Champreda:** Writing – review & editing, Validation, Supervision, Methodology, Funding acquisition, Conceptualization. **Atchara Paemaee:** Methodology, Formal analysis. **Navadol Laosiripojana:** Supervision, Funding acquisition.

## Declaration of Competing Interest

The authors declare no conflict of interest.

## Data Availability

All sample datasets are available on MassIVE at the University of California, San Diego Center for Computational Mass Spectrometry website (https://massive.ucsd.edu) with the following IDs: MSV000099285, MSV000099295, and MSV000099297.

## References

[1] Arya S.S., Venkatram R., More P.R., Vijayan P. (2021). The wastes of coffee bean processing for utilization in food: a review. J Food Sci Technol.

[2] Pakkir Shah A.K., Walter A., Ottosson F., Russo F., Navarro-Diaz M., Boldt J. (2024). Statistical analysis of feature-based molecular networking results from non-targeted metabolomics data. Nat Protoc 2024 201.

[3] Lee Y.-G., Cho E.-J., Maskey S., Nguyen D.-T., Bae H.-J. (2023). Value-Added Products from Coffee Waste: A Review. Molecules.

[4] Nguyen V., Taine E.G., Meng D., Cui T., Tan W. (2024). Chlorogenic acid: a systematic review on the biological functions, mechanistic actions, and therapeutic potentials. Nutrients.

[5] Franca A.S., Oliveira L.S. (2022). Potential uses of spent coffee grounds in the food industry. Foods.

[6] Johnson K., Liu Y., Lu M. (2022). A review of recent advances in spent coffee grounds upcycle technologies and practices. Front Chem Eng.

[7] Jeszka-Skowron M., Sentkowska A., Pyrzyńska K., De Peña M.P. (2016). Chlorogenic acids, caffeine content and antioxidant properties of green coffee extracts: influence of green coffee bean preparation. Eur Food Res Technol.

[8] Rojas-González A., Figueroa-Hernández C.Y., González-Rios O., Suárez-Quiroz M.L., González-Amaro R.M., Hernández-Estrada Z.J. (2022). Coffee Chlorogenic acids incorporation for bioactivity enhancement of foods: a review. Molecules.

[9] Vignoli J.A., Bassoli D.G., Benassi M.T. (2011). Antioxidant activity, polyphenols, caffeine and melanoidins in soluble coffee: the influence of processing conditions and raw material. Food Chem.

[10] Nzekoue F.K., Angeloni S., Navarini L., Angeloni C., Freschi M., Hrelia S. (2020). Coffee silverskin extracts: Quantification of 30 bioactive compounds by a new HPLC-MS/MS method and evaluation of their antioxidant and antibacterial activities. Food Res Int.

[11] Bouhzam I., Cantero R., Margallo M., Aldaco R., Bala A., Fullana-i-Palmer P. (2023). Extraction of Bioactive Compounds from Spent Coffee Grounds Using Ethanol and Acetone Aqueous Solutions. Foods.

[12] Fernandes F., Delerue-Matos C., Grosso C. (2025). Valorization of spent coffee grounds: comparing phenolic content and antioxidant activity in solid-liquid vs. subcritical water extraction methods. Biol Life Sci Forum.

[13] Vandeponseele A., Draye M., Piot C., Bernard D., Fanget P., Chatel G. (2022). Supercritical carbon dioxide in presence of water for the valorization of spent coffee grounds: optimization by response surface methodology and investigation of caffeine extraction mechanism. Foods.

[14] Nothias L.F., Petras D., Schmid R., Dührkop K., Rainer J., Sarvepalli A. (2020). Feature-based molecular networking in the GNPS analysis environment. Nat Methods 2020 179.

[15] Wang M., Carver J.J., Phelan V.V., Sanchez L.M., Garg N., Peng Y. (2016). Sharing and community curation of mass spectrometry data with global natural products social molecular networking. Nat Biotechnol.

[16] Vincenti F., Montesano C., Di Ottavio F., Gregori A., Compagnone D., Sergi M. (2020). Molecular networking: a useful tool for the identification of new psychoactive substances in seizures by LC–HRMS. Front Chem.

[17] Salviati E., Sommella E., Carrizzo A., Di Sarno V., Bertamino A., Venturini E. (2021). Characterization of phase I and phase II metabolites of hop (Humulus lupulus L.) bitter acids: In vitro and in vivo metabolic profiling by UHPLC-Q-Orbitrap. J Pharm Biomed Anal.

[18] Ma D., Pang Y., Xie R., Luo J., Xiao S., Wang J. (2024). Unveiling metabolite network dynamics during Pu-erh tea storage via non-targeted metabolomics. LWT.

[19] Montis A., Souard F., Delporte C., Stoffelen P., Stévigny C., Van Antwerpen P. (2022). Targeted and untargeted mass spectrometry-based metabolomics for chemical profiling of three coffee species. Molecules.

[20] Le D.D., Yu S., Dang T., Lee M. (2023). Molecular networking and bioassay-guided preparation and separation of active extract and constituents from Vicia tenuifolia Roth. Antioxidants.

[21] Li Y., Shahkoomahally S., Yang T., Chen P., Zhang M., Sun J. (2025). Metabolomics and molecular networking approach for exploring the effect of light intensity and quality on the chemical profile and accumulation of glucosinolates in broccoli microgreen. J Agric Food Chem.

[22] Hussen E.M., Endalew S.A. (2023). In vitro antioxidant and free-radical scavenging activities of polar leaf extracts of Vernonia amygdalina. BMC Complement Med Ther.

[23] Schmid R., Heuckeroth S., Korf A., Smirnov A., Myers O., Dyrlund T.S. (2023). Integrative analysis of multimodal mass spectrometry data in MZmine 3. Nat Biotechnol 2023 414.

[24] Shannon P., Markiel A., Ozier O., Baliga N.S., Wang J.T., Ramage D. (2003). Cytoscape: a software environment for integrated models of biomolecular interaction networks. Genome Res.

[25] Dührkop K., Fleischauer M., Ludwig M., Aksenov A.A., Melnik A.V., Meusel M. (2019). SIRIUS 4: a rapid tool for turning tandem mass spectra into metabolite structure information. Nat Methods.

[26] Al-Shemmeri M., Fryer P., Farr R., Lopez-Quiroga E. (2024). Development of coffee bean porosity and thermophysical properties during roasting. J Food Eng.

[27] Schenker S., Handschin S., Frey B., Perren R., Escher F. (2000). Pore structure of coffee beans affected by roasting conditions. J Food Sci.

[28] Somnuk K., Eawlex P., Prateepchaikul G. (2017). Optimization of coffee oil extraction from spent coffee grounds using four solvents and prototype-scale extraction using circulation process. Agric Nat Resour.

[29] Efthymiopoulos I., Hellier P., Ladommatos N., Russo-Profili A., Eveleigh A., Aliev A. (2018). Influence of solvent selection and extraction temperature on yield and composition of lipids extracted from spent coffee grounds. Ind Crops Prod.

[30] Arya S.S., Venkatram R., More P.R., Vijayan P. (2021). The wastes of coffee bean processing for utilization in food: a review. J Food Sci Technol.

[31] Mussatto S.I., Ballesteros L.F., Martins S., Teixeira J.A. (2011). Extraction of antioxidant phenolic compounds from spent coffee grounds. Sep Purif Technol.

[32] Coelho J.P., Filipe R.M., Paula Robalo M., Boyadzhieva S., Cholakov G.S., Stateva R.P. (2020). Supercritical CO_2_ extraction of spent coffee grounds. Influence of co-solvents and characterization of the extracts. J Supercrit Fluids.

[33] Badmos S., Lee S.H., Kuhnert N. (2019). Comparison and quantification of chlorogenic acids for differentiation of green Robusta and Arabica coffee beans. Food Res Int.

[34] Bevilacqua E., Cruzat V., Singh I., Rose’Meyer R.B., Panchal S.K., Brown L. (2023). The potential of spent coffee grounds in functional food development. Nutr 2023 Vol 15 Page 994.

[35] Kocadağlı T., Gökmen V. (2016). Effect of roasting and brewing on the antioxidant capacity of espresso brews determined by the QUENCHER procedure. Food Res Int.

[36] Maiyah N., Kerdpiboon S., Supapvanich S., Kerr W.L., Sriprom P., Chotigavin N. (2025). Recovering bioactive compounds and antioxidant capacity of medium roasted spent coffee grounds through varied hydrothermal brewing cycles. J Agric Food Res.

[37] Gloess A.N., Schönbächler B., Klopprogge B., D’Ambrosio L., Chatelain K., Bongartz A. (2013). Comparison of nine common coffee extraction methods: instrumental and sensory analysis. Eur Food Res Technol.

[38] Custodio-Mendoza J.A., Pokorski P., Aktaş H., Napiórkowska A., Kurek M.A. (2024). Advances in chromatographic analysis of phenolic phytochemicals in foods: bridging gaps and exploring new horizons. Foods.

[39] Nerurkar P.V., Yokoyama J., Ichimura K., Kutscher S., Wong J., Bittenbender H.C. (2023). Medium roasting and brewing methods differentially modulate global metabolites, lipids, biogenic amines, minerals, and antioxidant capacity of Hawai‘i-Grown Coffee (Coffea arabica). Metabolites.

[40] Fuller M., Rao N.Z. (2017). The effect of time, roasting temperature, and grind size on caffeine and chlorogenic acid concentrations in cold brew coffee. Sci Rep 2017 71.

[41] Farag M.A., Zayed A., Sallam I.E., Abdelwareth A., Wessjohann L.A. (2022). Metabolomics-based approach for coffee beverage improvement in the context of processing, brewing methods, and quality attributes. Foods.

[42] Lyrio M.V.V., Debona D.G., Feu A.E., dos Santos N.A., Gonçalves A. da S., Kuster R.M. (2025). LC-ESI(±)-LTQ MSn-based metabolomic profiling of coffee: fragmentation pathways for identification of major polar compounds. J Am Soc Mass Spectrom.

