# Supplementary material for: Multisolvent metabolite profiling of coffee waste by UHPLC-HRMS/MS and molecular networking
Source: Comput Struct Biotechnol J. 2025 Oct 30;27:5116–28. doi: 10.1016/j.csbj.2025.10.060 (PMC12663856; doi:10.1016/j.csbj.2025.10.060)
Supplement: Supplementary file 1 — Supplementary material [file mmc1.docx]

**Supplementary Material**

**Multisolvent Metabolite Profiling of Coffee Waste by UHPLC-HRMS/MS and Molecular Networking**

Surachet Soontontaweesub^a,b^, Rungvigrai Lertsuwan^a,b^, Thapanee Pruksatrakul^a,b^, Atchara Paemaee^c^, Verawat Champreda^b*^, and Navadol Laosiripojana^a^

^a^The Joint Graduate School for Energy and Environment (JGSEE), King Mongkut's University of Technology Thonburi, Prachauthit Road, Bangmod, Bangkok 10140, Thailand

^b^Biorefinery Technology and Bioproducts Research Group, National Center for Genetic Engineering and Biotechnology (BIOTEC), 113 Thailand Science Park, Phaholyothin Road, Khlong Luang, Pathumthani 12120, Thailand

^c^Food Biotechnology Research Team, Functional Ingredients and Food Innovation Research Group, National Center for Genetic Engineering and Biotechnology (BIOTEC), 113 Thailand Science Park, Phaholyothin Road, Khlong Luang, Pathumthani 12120, Thailand

*Corresponding author: Tel: +66-2564-6700 (ext 3446); Email: verawat@biotec.or.th

**
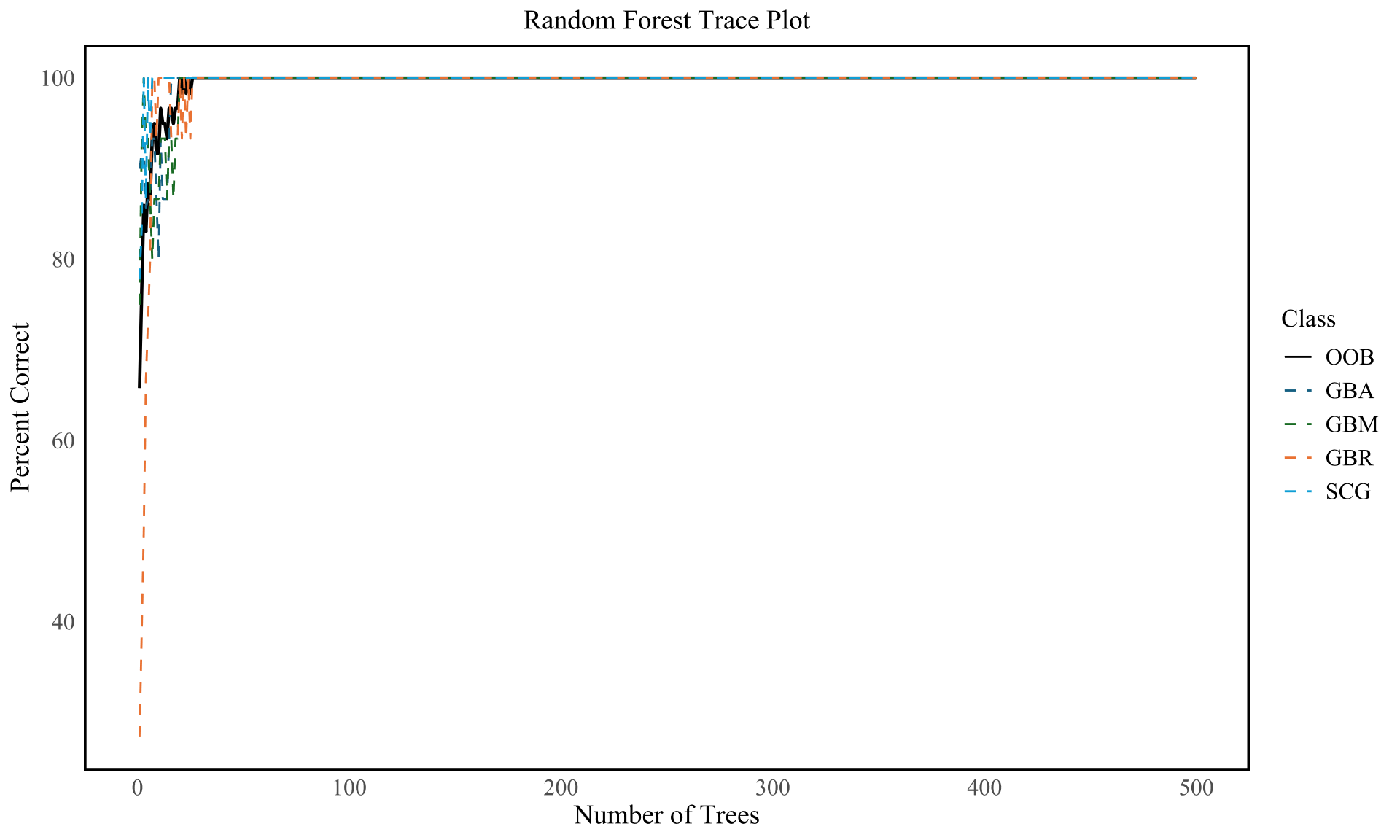
**

**Figure S1** Out-of-bag error curve of random forest classification in negative mode for raw material


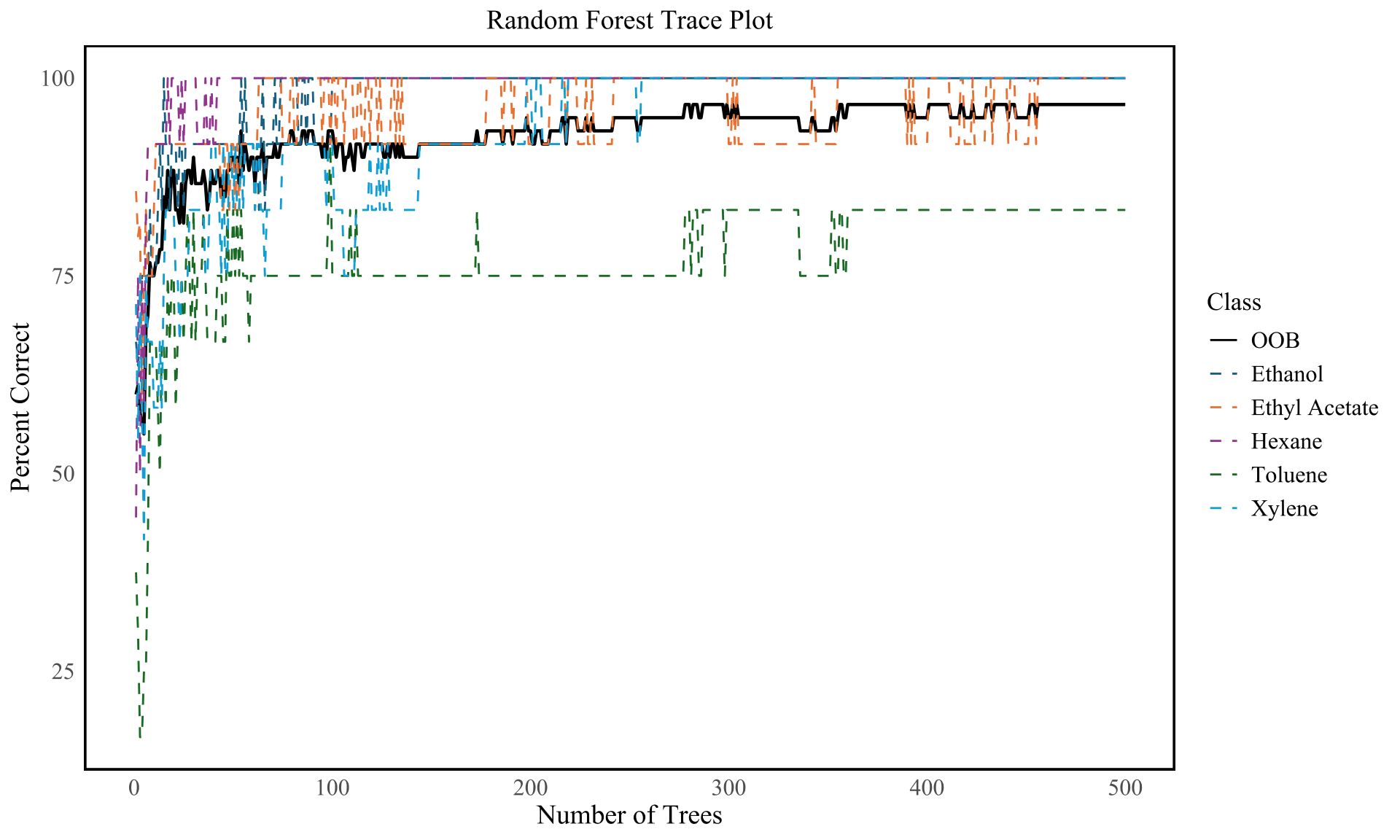


**Figure S2** Out-of-bag error curve of random forest classification in negative mode for solvent

**
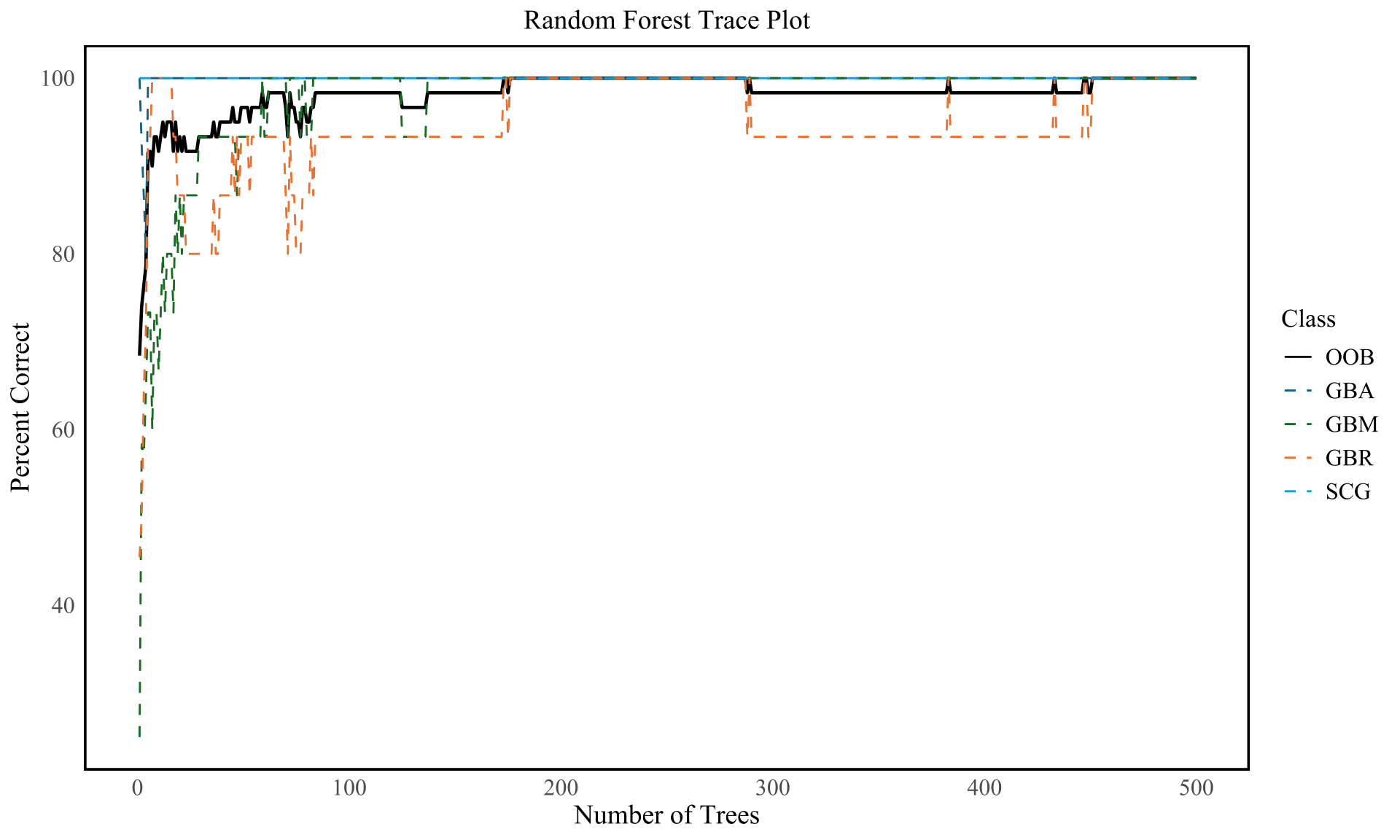
**

**Figure S3** Out-of-bag error curve of random forest classification in negative mode for raw material.


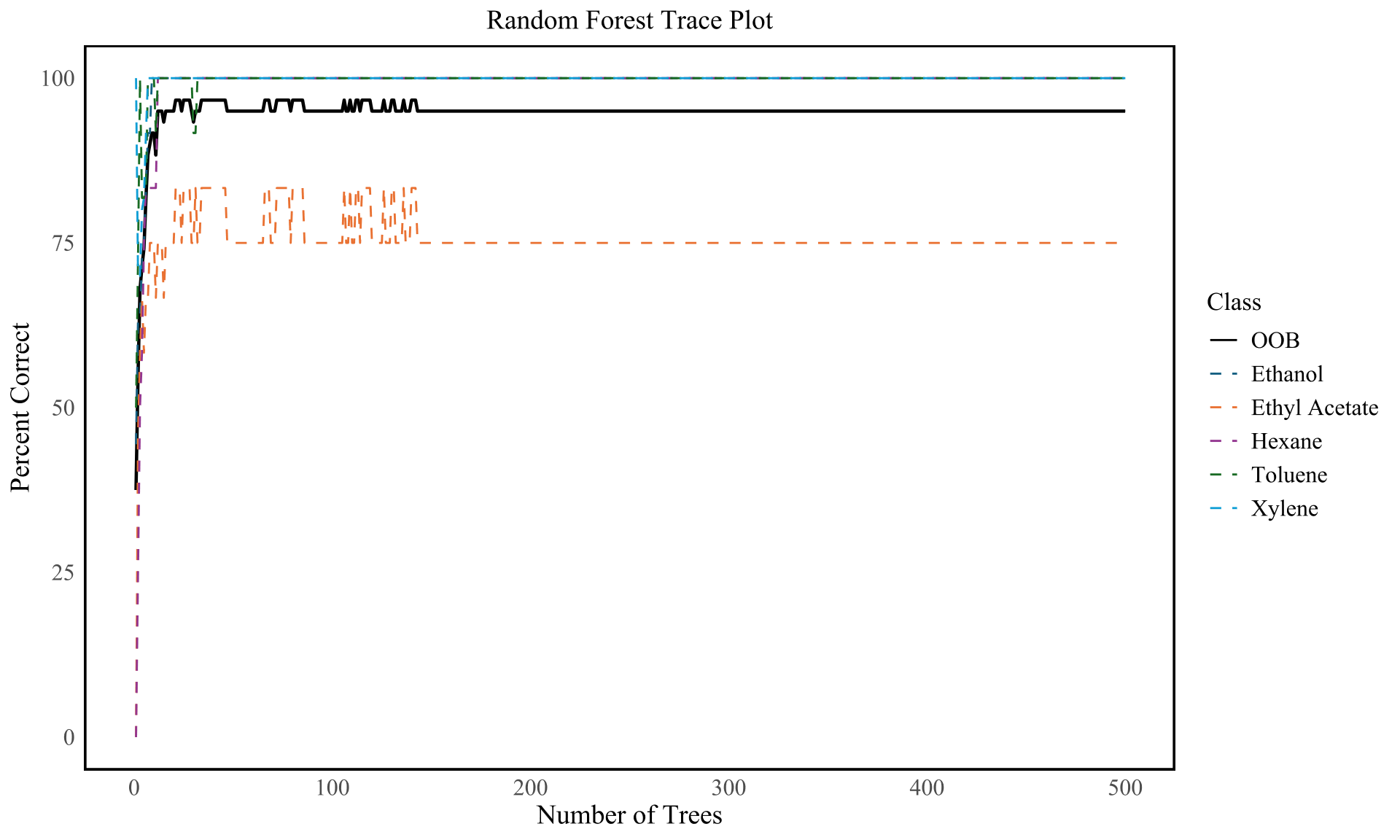


**Figure S4** Out-of-bag error curve of random forest classification in positive mode for solvent.


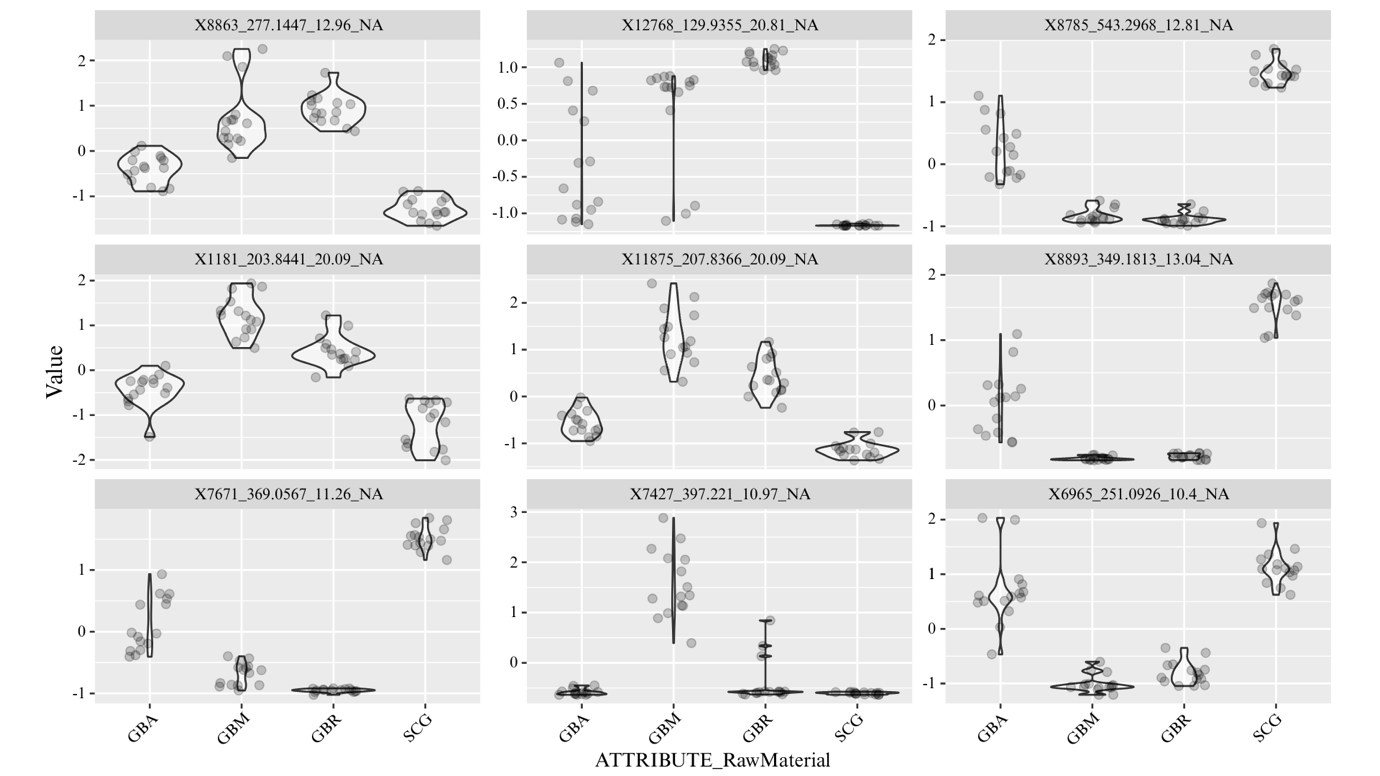


**Figure S5** Random Forest feature importance plot of model classification in negative mode for raw material. Each panel shows the normalized abundance of the top-ranked variables (mean decrease accuracy) identified by the RF model.


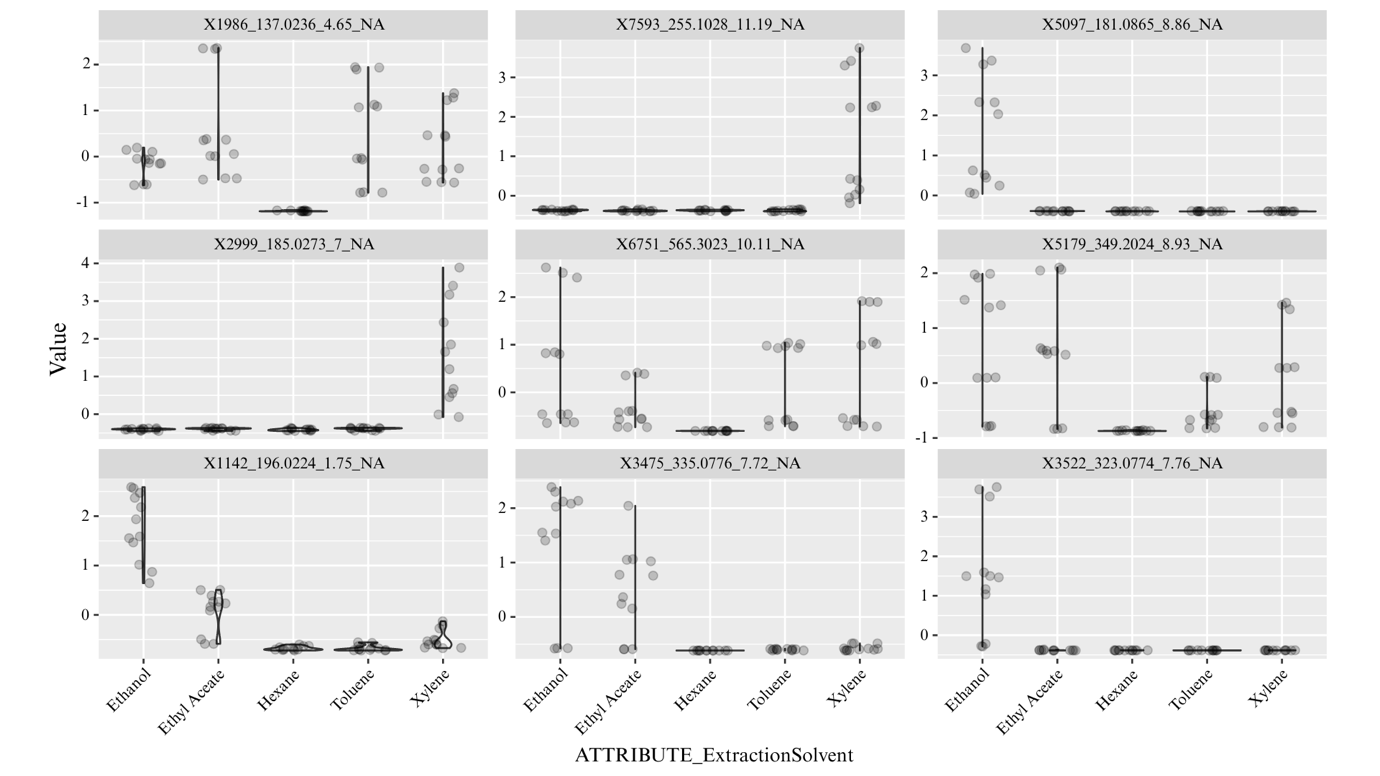


**Figure S6** Random Forest feature importance plot of model classification in negative mode for solvent. Each panel shows the normalized abundance of the top-ranked variables (mean decrease accuracy) identified by the RF model.


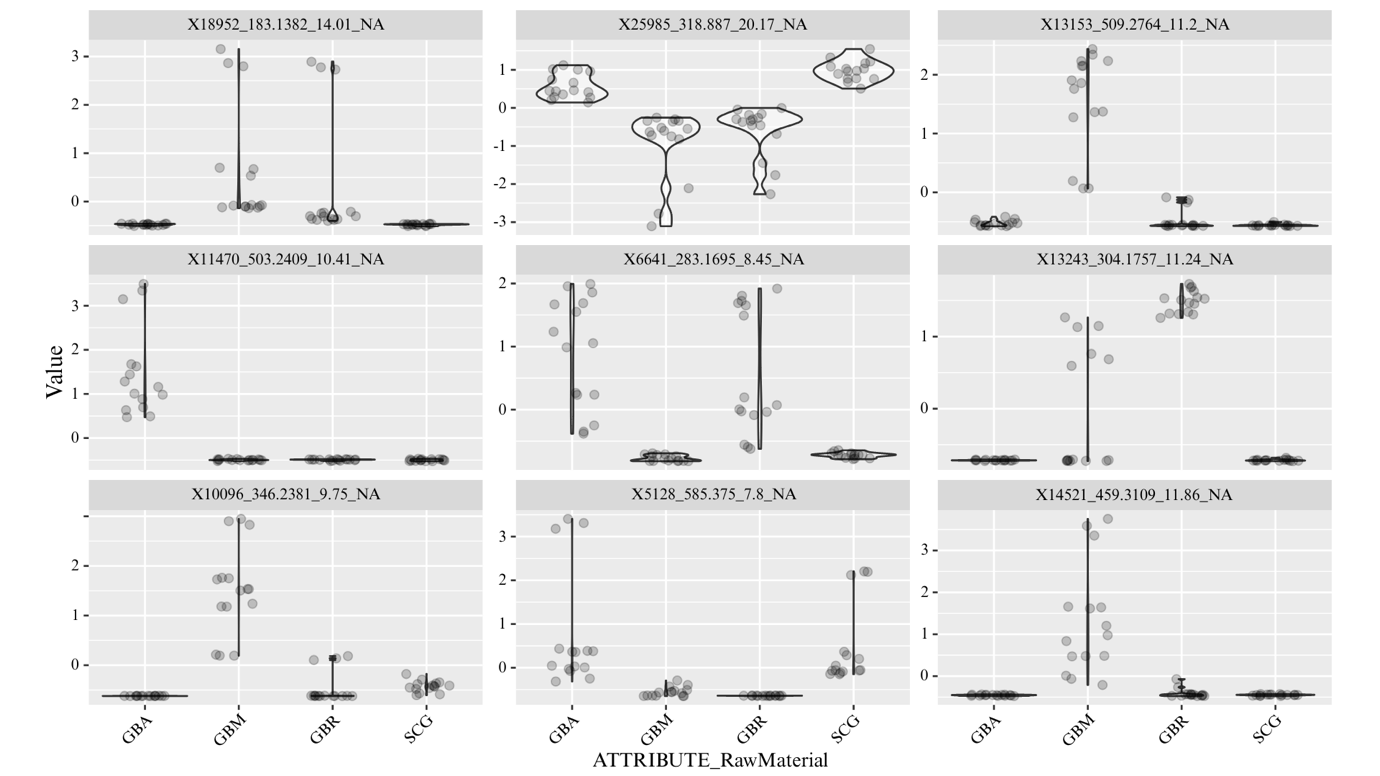


**Figure S7** Random Forest feature importance plot of model classification in negative mode for raw material. Each panel shows the normalized abundance of the top-ranked variables (mean decrease accuracy) identified by the RF model.


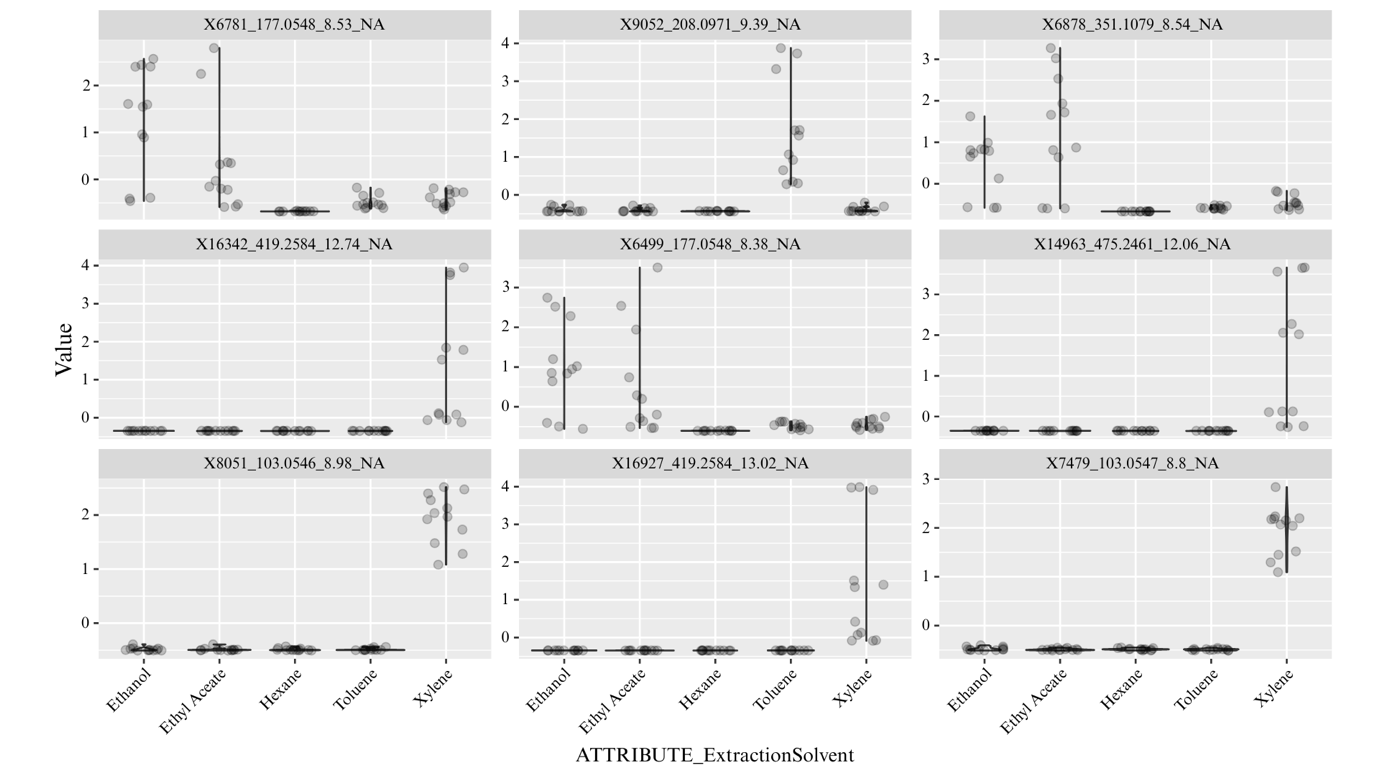


**Figure S8** Random Forest feature importance plot of model classification in positive mode for solvent. Each panel shows the normalized abundance of the top-ranked variables (mean decrease accuracy) identified by the RF model.

**Table S1** Confusion matrix of Random Forest classification in negative mode for raw material.

|  | GBA | GBM | GBR | SCG | Pct | LCI_0.95 | UCI_0.95 |
| --- | --- | --- | --- | --- | --- | --- | --- |
| GBA | 15 | 0 | 0 | 0 | 100.0 | 78.2 | 100.0 |
| GBM | 0 | 15 | 0 | 0 | 100.0 | 78.2 | 100.0 |
| GBR | 0 | 0 | 15 | 0 | 100.0 | 78.2 | 100.0 |
| SCG | 0 | 0 | 0 | 15 | 100.0 | 78.2 | 100.0 |
| Overall | NA | NA | NA | NA | 100.0 | 94.0 | 100.0 |

GBA: Green Bean Arabica, GBM: Green Bean Mix, GBR: Green Bean Robusta, SCG: Spent Coffee Ground, NA: Not Available, Pct.: Percent Correct, LCI: Lower Confidence Interval, and UCI: Upper Confidence Interval.

**Table S2** Confusion matrix of Random Forest classification in negative mode for solvent.

|  | Ethanol | Ethyl  Acetate | Hexane | Toluene | Xylene | Pct | LCI_0.95 | UCI_0.95 |
| --- | --- | --- | --- | --- | --- | --- | --- | --- |
| Ethanol | 12 | 0 | 0 | 0 | 0 | 100.0 | 73.5 | 100.0 |
| Ethyl Acetate | 0 | 12 | 0 | 0 | 0 | 100.0 | 73.5 | 100.0 |
| Hexane | 0 | 0 | 12 | 0 | 0 | 100.0 | 73.5 | 100.0 |
| Toluene | 0 | 0 | 2 | 10 | 0 | 83.3 | 51.6 | 97.9 |
| Xylene | 0 | 0 | 0 | 0 | 12 | 100.0 | 73.5 | 100.0 |
| Overall | NA | NA | NA | NA | NA | 96.7 | 88.5 | 99.6 |

NA: Not Available, Pct.: Percent Correct, LCI: Lower Confidence Interval and UCI: Upper Confidence Interval

**Table S3** Confusion matrix of Random Forest classification in positive mode for raw material.

|  | GBA | GBM | GBR | SCG | Pct | LCI_0.95 | UCI_0.95 |
| --- | --- | --- | --- | --- | --- | --- | --- |
| GBA | 15 | 0 | 0 | 0 | 100.0 | 78.2 | 100.0 |
| GBM | 0 | 15 | 0 | 0 | 100.0 | 78.2 | 100.0 |
| GBR | 0 | 0 | 15 | 0 | 100.0 | 78.2 | 100.0 |
| SCG | 0 | 0 | 0 | 15 | 100.0 | 78.2 | 100.0 |
| Overall | NA | NA | NA | NA | 100.0 | 94.0 | 100.0 |

GBA: Green Bean Arabica, GBM: Green Bean Mix, GBR: Green Bean Robusta, SCG: Spent Coffee Ground, NA: Not Available, Pct.: Percent Correct, LCI: Lower Confidence Interval, and UCI: Upper Confidence Interval.

**Table S4** Confusion matrix of Random Forest classification in positive mode for solvent.

|  | Ethanol | Ethyl  Acetate | Hexane | Toluene | Xylene | Pct | LCI_0.95 | UCI_0.95 |
| --- | --- | --- | --- | --- | --- | --- | --- | --- |
| Ethanol | 12 | 0 | 0 | 0 | 0 | 100.0 | 73.5 | 100.0 |
| Ethyl Acetate | 0 | 9 | 0 | 3 | 0 | 75.0 | 42.8 | 94.5 |
| Hexane | 0 | 0 | 12 | 0 | 0 | 100.0 | 73.5 | 100.0 |
| Toluene | 0 | 0 | 0 | 12 | 0 | 100.0 | 73.5 | 100.0 |
| Xylene | 0 | 0 | 0 | 0 | 12 | 100.0 | 73.5 | 100.0 |
| Overall | NA | NA | NA | NA | NA | 95.0 | 86.1 | 99.0 |

NA: Not Available, Pct.: Percent Correct, LCI: Lower Confidence Interval and UCI: Upper Confidence Interval
